# A Method for the Observation of the Primo Vascular System in the Thoracic Duct of a Rat

**DOI:** 10.1155/2013/536560

**Published:** 2013-06-12

**Authors:** Sungha Kim, Sharon Jiyoon Jung, Sang Yeon Cho, Yoon Kyu Song, Kwang-Sup Soh, Sungchul Kim

**Affiliations:** ^1^Department of Acupuncture & Moxibustion, Wonkwang University, Gwangju Medical Hospital, Gwangju 503-310, Republic of Korea; ^2^Nano Primo Research Center, Advanced Institute of Convergence Technology, Seoul National University, Suwon 443-270, Republic of Korea; ^3^Graduate School of Convergence Science Technology, Seoul National University, Suwon 443-270, Republic of Korea

## Abstract

Even though the primo vascular system (PVS) has been observed in large caliber lymph vessels by several independent teams, the presence of the PVS in the thoracic duct has been reported by only one team, probably because reproducing the experiment is technically difficult. This brief report presents a new, relatively straightforward method, which is a simple modification of the previous method of dye injection into the lumbar node, to observe the PVS in a thoracic duct of a rat by injecting Alcian blue into the renal node. When this new method was applied to a rat, the branching of the primo vessel in the thoracic duct was clearly displayed. Thus, this new method is expected to extend the network of the PVS from abdominal lymph ducts to thoracic ones.

## 1. Introduction

Observation of the primo vascular system (PVS) in the thoracic lymph ducts of rodents was reported earlier [1], but reproducing that experiment was difficult; thus, no further work by other independent groups has been performed. In this brief report, we present a different method to observe the PVS in the thoracic duct of a rat.

Our method is to inject Alcian blue staining dye into the renal lymph node near the kidney. In fact, dye injection techniques have been used to find the PVS in the lymph vessel between the lumbar and the mesenteric nodes in rabbits [2–5] and rats [6, 7]. In these previous experiments, the dye was injected into the lumbar nodes, and the stained primo vessel was traced only up to the diaphragm because the researchers were trying to observe the PVS under *in vivo* conditions. In the current work, which is still in progress, in order to expand the range of PVS observations, we opened the thoracic cavity thirty minutes after injecting the dye into the renal lymph node, and we were able to observe the PVS in the thoracic duct *in situ* but not *in vivo*.

Recently, the primo nodes in the lymph vessels of rats were found to be enriched with immune cells, such as macrophages, mast cells, and neutrophils. Thus, the PVS might play an important role in the immune mechanism [8]. Therefore, extending the network of the PVS as much as possible to obtain larger numbers of specimens would seem to be an appropriate endeavor. The current brief report is intended to serve that purpose.

## 2. Materials and Methods

### 2.1. Animals

For the laboratory animals, male Sprague-Dawley (SD) rats (*n* = 7, 9 weeks old) were purchased from DooYeol Biotech (Seoul, Korea). Rats were kept in constant temperature and humidity conditions (23°C, relative humidity: 60%) with a 12/12 light/dark cycle and were provided with water and commercial rat chow *ad libitum*. The procedures involving the animals and their care were in full compliance with current international laws and policies (Guide for the Care and Use of Laboratory Animals, National Academy Press, 1996).

### 2.2. Surgery and Alcian Blue Injection

We anesthetized the rats by using an intramuscular injection of a mixed solution of 1.5 g/kg of urethane (or 50 mg/kg of Zoletil) and 1 mL of Rompun. With surgical scissors, we incised the outermost skin along the linea alba of the abdomen from the navel down to the symphysis pubis and again up to the ensisternum. Then, we cut the straight muscle of the abdomen to expose the internal organs and moved the organs to the side for observation of the target renal lymph node.

The Alcian blue solution was prepared from 0.1 g of Alcian blue (Sigma-Aldrich, St. Louis, MO, USA) dissolved in 10 mL of phosphate-buffered saline (PBS, pH 7.4) and was filtered by using a 0.22 *μ*m syringe filter (Millipore, Bedford, MA, USA) with a 10 mL syringe (BD, Franklin Lakes, NJ, USA). After the rat's abdomen had been incised along the linea alba, the Alcian blue solution was preheated to 37°C in a water bath and was prepared for injection into a renal lymph node. After the staining dye had been injected into the left renal node, in order to promote the natural circulation of the lymph fluid inside the ducts for the purpose of washing the staining dye, the internal organs that had been moved to the side were replaced in their original positions, and the abdominal skin was closed with forceps. This step was necessary to raise the body temperature. The rats were sacrificed with an intracardiac injection of 0.7 mL urethane at about 30 minutes after the Alcian blue staining dye injection. The thorax of the rat was opened along the right side of the sternum, and the heart and the lungs were removed to observe the thoracic duct. For clear observation, careful incision and removal were necessary to minimize bleeding.

For *in situ* observation of the primo vessel inside the thoracic duct, we used a stereomicroscope (SZX12, Olympus, Tokyo, Japan). We put the rat in 10% neutral buffered formalin solution for one day and then washed it for two hours with tap water. We took the PVS specimen extracted from the thoracic duct, put it on a microscope slide, and examined it with a phase contrast microscope after staining.

### 2.3. Staining and Microscopy

We applied 4′,6′-diamidino-2-phenylindole (DAPI) and phalloidin reagents for staining of nuclei and f-actins in the cells, respectively. After a 1-hour DAPI (Invitrogen, Prolong Gold Antifade Reagent with DAPI, St. Louis, MO, USA) staining, we washed the sample three times with PBS solution. The phalloidin (Invitrogen, Rhodamine Phalloidin, St. Louis, MO, USA) staining was done in the same way as the DAPI staining.

The prepared sample was investigated under a phase contrast microscope (Olympus Model number BX51, Tokyo, Japan) in order to observe the distributions of the nuclei and the f-actin of the primo vessels that had been stained with DAPI and phalloidin, respectively. Confocal laser scanning microscopy (CLSM; Nikon, C1 plus, Tokyo, Japan) was used to examine optical sections of the threadlike primo vessel.

## 3. Results

**Figure 1:**
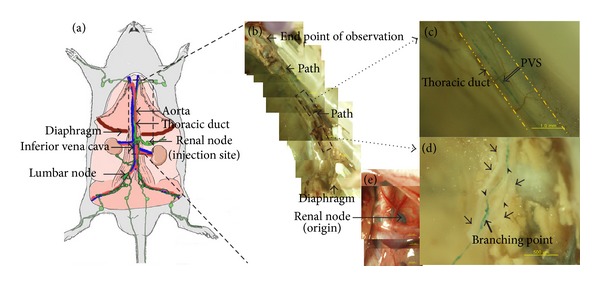
Anatomical location of the primo vascular system (PVS) in the thoracic duct of a rat. (a) Schematic anatomical view of the rat. Large-caliber lymph vessels are depicted with green curves and large arteries and veins with red and blue curves, respectively. (b) Stereomicroscopic image of the thoracic duct indicated with arrows. (c) Magnified view showing the primo vessel (PVS, open arrow) in the thoracic duct (arrow and two dotted lines). (d) Another magnified view showing branched primo vessels (arrow heads) in the thoracic duct (arrows). (e) Stereomicroscopic image of the left renal node (arrows) under the diaphragm. This is the injection site which became blue due to Alcian blue. The colors of panels (b) and (e) are different because (b) was taken after NBF fixing of the euthanized rat, while (e) was taken *in vivo *immediately after injection.

The thoracic duct is a continuation of the largest-caliber abdominal lymph vessel along the caudal vena cava (Figure 1(a)). The Alcian blue was injected into the renal node, flowed into the thoracic duct, and stained the PVS floating in the thoracic duct as indicated in Figure 1(b). A magnified view of the thoracic duct (dotted line) and the primo vessel (blue curve) is given in Figure 1(c). The primo vessel was a continuous thread from the thread in the abdominal lymph vessel below the diaphragm. There were several branches and rejoins of the thoracic lymph duct, and one of them is shown in Figure 1(d). The primo vessel floating inside the thoracic lymph duct also branched and rejoined (blue curves). The thoracic lymph duct before the branching point was stripped, and a blue primo vessel was exposed.

**Figure 2:**
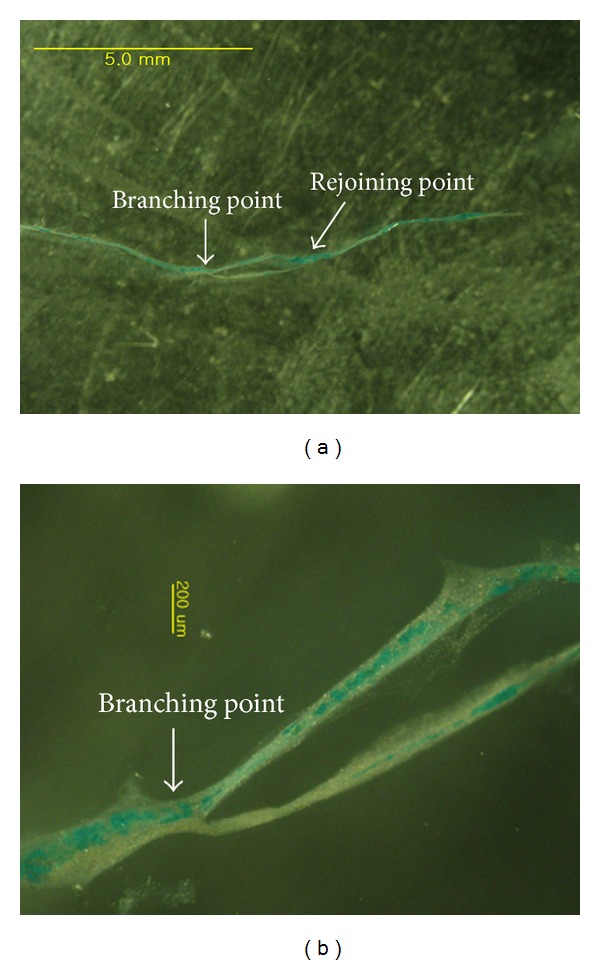
Stereomicroscopic images of the branched primo vessels (PVS) of Figure 1(d), which were extracted from the thoracic duct and put on a slide. Panel (b) is a magnified view of (a).

**Figure 3:**
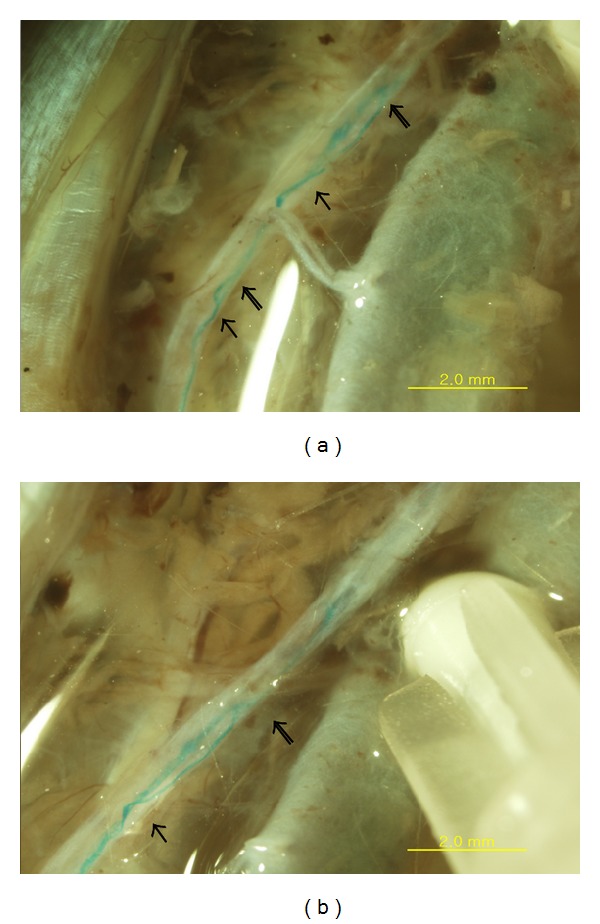
Stereomicroscopic *in situ* image of a branched and rejoined primo vascular system (blue stained cure) in the thoracic duct of another subject rat. (a) Two regions of branching (arrows) and rejoining (open arrows) are observed, and the upper one is magnified in (b).

The branched primo vessel was extracted from the duct and put on a slide, as shown in Figure 2(a). A magnified view of the branch showed only partial Alcian blue staining (Figure 2(b)), and the reason for that partial staining is not yet understood. The branching of the thoracic duct and the primo vessel in it was a common phenomenon, as shown in Figure 3, where branching and rejoining were seen to occur twice. The image in panel (b) is a magnified view of the image in panel (a), which was taken *in situ* with a stereomicroscope.

**Figure 4:**
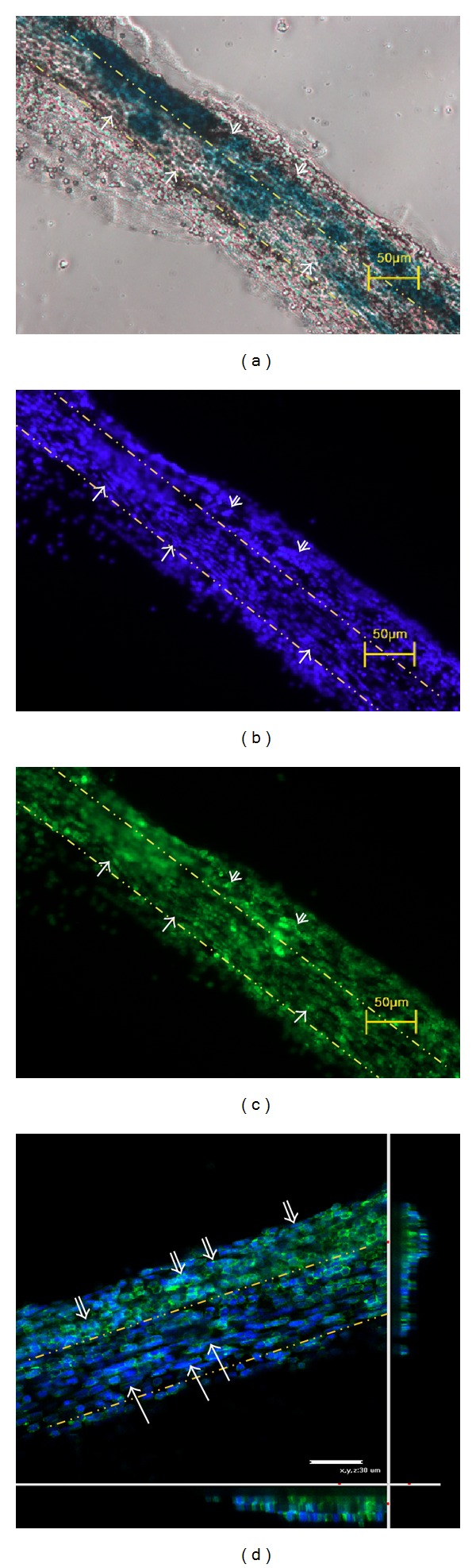
Morphological features of a primo vessel (inside the two dotted lines) in a thoracic duct. (a) Phase contrast microscopic image of the primo vessel. (b) Rod-shaped nuclei longitudinally arranged along the primo vessel (inside the yellow-lined region) stained with DAPI. (c) f-actin signals in the cell plasma of the primo vessel stained with phalloidin. (d) Confocal laser scanning microscopic (CLSM) image of the f-actin signals (green) and the nuclei (blue) of the primo vessel. The under and the side panels show cross-sectional views. Rod-shaped nuclei (arrows) are longitudinally arranged along the primo vessel, and lymphocytes (double arrows) are aggregated around the primo vessel.

In order to confirm that the stained threadlike structure was a primo vessel, we applied the previously established simple criteria of DAPI staining of nuclei and phalloidin staining of f-actins [9]. As shown in the DAPI image, the alignment of rod-shaped nuclei in parallel with the primo vessel was in good agreement with the criteria (Figure 4(b)). Notice that the rod-shaped nuclei were present only inside the region defined by two broken lines. Outside the region, round-shaped nuclei, which are aggregated lymphocytes, were observed. The phase contrast image more clearly showed the aggregated round-shaped lymphocytes scattered around the primo vessel whose boundaries were indicated by the two broken lines (Figure 4(a)). Also, the distribution of the f-actins in the cytoplasm was in agreement with that for a typical primo vessel and was distinctively different from that for a lymph or a blood vessel (Figure 4(c)); that is, the phalloidin signals were aligned along the vessel. The confocal laser scanning microscope image more clearly showed the rod-shaped nuclei (blue color) (Figure 4(d)). 

The lengths of the nuclei were 8.3–14 *μ*m, as expected from Bong-Han Kim's work [10]. Another important morphological datum is the diameter of the primo vessel, and it was, on average, 62 ± 28 *μ*m. The morphological size data for the primo vessels from the thoracic ducts of the subject rats are given in Table 1.

## 4. Discussion

In this brief report, we presented a repeatable method for observing the PVS in the thoracic duct of a rat. Even though the thoracic duct is the largest-caliber lymph vessel, the primo vessel found in this duct is not necessarily much thicker than those found in less-large-caliber lymph vessels. The average diameter of the primo vessels in our case was 61.8 ± 28.3 *μ*m, while the average value of the primo vessels found in the lymph vessels in abdominal cavities was 52 ± 30 *μ*m (rat) [6]. This uniform size was also in agreement with the sizes of the primo vessels found in blood vessels and on the surfaces of internal organs [9]. In our case, the diameter was somewhat larger because of the aggregation of lymphocytes around the primo vessel, as seen in Figure 4. A future task is to remove these aggregated lymphocytes to obtain pure specimens.

This work is the first report on a primo vessel seen in the whole thoracic duct in the longitudinal direction, although a cross-sectional image was presented earlier [1]. In addition, in this work, the branching and the rejoining of the thoracic duct and its associated primo vessel, as mentioned in Bong-Han Kim's work [10], were first demonstrated.

The primo vessel specimen taken from the thoracic duct showed the characteristic hallmarks of a primo vessel; namely, the DAPI images of the shapes and the distribution of nuclei and the phalloidin images of f-actins. We did not address further histological analysis in this brief report partially because the results for the basic H&E-stained specimen have already been reported [1] and partially because an immunohistochemical examination of its extended part in the abdominal lymph vessels was thoroughly done in another work [8]. Another reason was that our main purpose was to develop a repeatable method that could be reproduced by other independent groups. We were able to demonstrate that the method of injecting Alcian blue into a lumbar node could be modified to inject it into a renal node to detect a primo vessel in the thoracic duct.

## Figures and Tables

**Table 1:** Morphological size data for the primo vessels from the thoracic ducts of seven male, nine-week-old rats.

Subject	Diameter of lymph vessel (mm)	Diameter of primo vessel (*μ*m)
1	1.0	58.8
2	0.4	88.2
3	0.9	85.0
4	0.3	73.5
5	1.0	72.1
6	0.5	36.4
7	0.4	18.3
Average ± S.D.	0.6 ± 0.3	61.8 ± 28.3
